# A novel series of compositionally biased substitution matrices for comparing *Plasmodium *proteins

**DOI:** 10.1186/1471-2105-9-236

**Published:** 2008-05-16

**Authors:** Kevin Brick, Elisabetta Pizzi

**Affiliations:** 1 Dipartimento di Malattie Infettive, Parassitarie ed Immunomediate – Istituto Superiore di Sanità, Viale Regina Elena, 299 00161 Roma, Italy

## Abstract

**Background:**

The most common substitution matrices currently used (BLOSUM and PAM) are based on protein sequences with average amino acid distributions, thus they do not represent a fully accurate substitution model for proteins characterized by a biased amino acid composition. This problem has been addressed recently by adjusting existing matrices, however, to date, no empirical approach has been taken to build matrices which offer a substitution model for comparing proteins sharing an amino acid compositional bias. Here, we present a novel procedure to construct series of symmetrical substitution matrices to align proteins from similarly biased *Plasmodium *proteomes.

**Results:**

We generated substitution matrices by selecting from the BLOCKS database those multiple alignments with a compositional bias similar to that of *P. falciparum *and *P. yoelii *proteins. A novel 'fuzzy' clustering method was adopted to group sequences within these alignments, showing that this method retains more complete information on the amino acid substitutions when compared to hierarchical clustering. We assessed the performance against the BLOSUM62 series and showed that the usage of our matrices results in an improvement in the performance of BLAST database searches, greatly reducing the number of false positive hits. We then demonstrated applications of the use of novel matrices to improve the annotation of homologs between the two *Plasmodium *species and to classify members of the *P. falciparum *RIFIN/STEVOR family.

**Conclusion:**

We confirmed that in the case of compositionally biased proteins, standard BLOSUM matrices are not suited for optimal alignments, and specific substitution matrices are required. In addition, we showed that the usage of these matrices leads to a reduction of false positive hits, facilitating the automatic annotation process.

## Background

The most widely used series of substitution matrices, BLOSUM [1] and PAM [2] were developed using the same mathematical model which dictates that in a system where amino acids occur randomly, a matrix can be built in the log-odds form. While PAM matrices are based on an underlying evolutionary model, BLOSUM are not. However, it has been shown that matrices similar to BLOSUM can also be constructed using an improved theoretical model of amino acid substitution frequencies [3]. While PAM and BLOSUM matrices used different approaches to derive amino acid target frequencies, both were constructed to implicitly represent substitutions between proteins of average amino acid composition. Due to this implicit amino acid background, it has been demonstrated that such matrices are not optimal for comparing proteins with strongly biased amino acid distributions [4]. In fact, in these cases, the use of the BLOSUM62 matrix for BLAST searches can result in deviations from the expected extreme value distribution [5].

Several works have been carried out into developing matrices which more accurately reflect amino acid substitution frequencies between proteins with a compositional bias. Asymmetrical substitution matrices were constructed by Yu et al. [6] and Bastien et al. [7] in order to reflect the amino acid composition of both query and subject proteins in local alignments. In Yu et al., the BLOSUM62 matrix is adjusted for each pair of proteins to be aligned using the target amino acid frequencies of both query and subject proteins. With this adjustment, alignment of proteins with non-standard amino acid compositions showed closer agreement with predicted secondary structures and yielded higher bit scores when compared with alignments using unadjusted BLOSUM62 [8]. Bastien et al. [7] used a different approach on a comparison between proteins from an unbiased genome (*Arabidopsis thaliana*) and those from a biased one (*Plasmodium falciparum*). They introduced a method from information theory which allowed them to derive asymmetrical matrices iteratively from multiple alignments which included biased proteins.

The development of symmetrical substitution matrices has also been shown to be useful where proteins with similar bias are to be aligned. In Ng et al. [9], the PHAT matrices were constructed from carefully selected multiple alignments of hydrophobic and transmembrane regions, and were shown to be effective in improving local alignments of such regions. More recently, it was shown that by adjusting existing substitution matrices to compensate for the compositional bias in query proteins, better alignments of yeast glycoprotein low-complexity domains could be achieved [5]. Although these methods all address different aspects of the problem of compositional bias, the case of aligning related proteins with a similar extreme bias has yet to be extensively studied.

In this work we present a novel approach to generate a series of symmetrical matrices starting from a set of multiple alignments whose sequences reflect the compositional bias of proteins to be aligned. We focus on the proteins of two species of the genus *Plasmodium*. These protozoan parasites are the causal agents of malaria, for which the human forms result in over one million deaths annually [10]. In recent years a great effort has been undertaken to generate genomic sequence data for members of this genus, resulting in two complete genomes (*P. falciparum*, *P. yoelii*) [11,12] and many others with high sequence coverage (*P. knowlesi*, *P. vivax*, *P. chabaudi*, *P. berghei*, *P. reichenowi*). Analyses of complete genomes showed that *P. falciparum *and *P. yoelii *genomes are both characterized by an unusually high content of A+T nucleotides [11]. This results in a large proportion of proteins with a biased amino acid composition and a peculiar frequency of low-complexity domains [7,13,14].

In this work, we firstly show that the amino acid bias of *P. falciparum *and *P. yoelii *proteins is a proteome-wide phenomenon, not limited to low-complexity domains or to species specific proteins. Beginning from selected biased multiple alignments of the BLOCKS database [15,16], and applying a novel procedure to cluster sequences at different percentages of identity, we generate log-odds matrices with an intrinsic amino acid bias representative of the parasite proteins. Two series of matrices were constructed (CBM and CCF) according to criteria used to select multiple alignments in BLOCKS. The performances of CBM, CCF and BLOSUM matrices were assessed by means of BLAST database searches and it was observed that an improvement in the specificity and positive predictive values was obtained when CBM and CCF matrices were used. In particular, we show that the use of CBM and CCF matrices increases alignment scores for related proteins, and helps to filter out false positive hits. We then illustrate how our biased matrices may be used to improve ortholog detection within *Plasmodia *species and to distinguish members of *P. falciparum *multi gene families more clearly between sub-families.

## Results

### Scoring BLOCKS database

**Figure 1:**
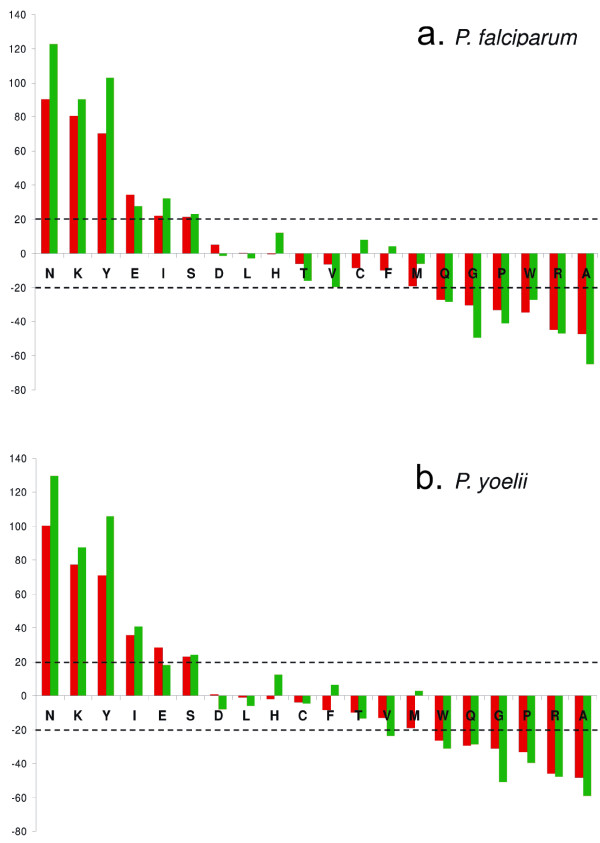
**Amino acid deviations from BLOSUM62 target frequencies**. We generated two datasets from each of the proteomes of *P. falciparum *and *P. yoelii*. One dataset contained proteins for which an annotated ortholog existed in a non-*Plasmodium *species (PfO, PyO), and the other contained proteins classified as hypothetical and which were not in the first dataset (PfH, PyH). The amino acid background frequencies implicit in the BLOSUM62 matrix were derived. *P. falciparum *and *P. yoelii *proteins in the four data sets PfO, PyO, PfH and PyH were filtered using the SEG algorithm (window size 24; locut 2.3; hicut 2.4), then for each data set the percent divergence of each amino acid from the BLOSUM62 background was calculated.

First of all, we analysed the compositional bias in *P. falciparum *and *P. yoelii *proteins to establish criteria with which to select blocks from the BLOCKS database [15,16]. For each organism, we selected those proteins for which an annotated ortholog was identified outside of the Plasmodia (PfO and PyO), and proteins classified as hypothetical without orthologs in other non-Plasmodia (PfH and PyH). The proteins of each dataset were filtered to eliminate low complexity sequences, then the amino acid content of these regions was calculated. We then assessed the deviation of each amino acid frequency from the BLOSUM62 compositional background (baseline y = 0 in figure 1). This allowed us to establish which amino acids were relatively over and under- represented in *Plasmodium *proteins, irrespective of their absolute abundance. For instance, Leucine and Asparagine are both frequently found in *Plasmodium *proteins [see Additional file 1], however, only Asparagine deviates strongly from the BLOSUM62 background. Results in figure 1 show that the compositional bias in *P. falciparum *and *P. yoelii *proteins are not solely a result of low-complexity regions [13,17], rather, that this feature is shared by complex sequences in both proteomes. Furthermore, while the compositional bias is more pronounced in those proteins currently annotated as parasite specific (PfH and PyH, green bars in figure 1), significant deviations from the BLOSUM62 baseline also are seen in proteins with orthologs in other species (PfO and PyO, red bars in figure 1). It is possible that the more pronounced bias for the PfH and PyH proteins is a consequence of current comparative methods not being optimised to assign orthologs to strongly biased proteins. Irrespectively, the bias observed in the high complexity domains of all parasite proteins validates the necessity to develop specific substitution matrices.

Amino acids that contributed most to the bias were identified as those which deviated by more than 20% from the baseline in at least one of the two datasets (PfO, PfH and PyO, PyH) both in *P. falciparum *and in *P. yoelii *(Asn, Lys, Tyr, Glu, Ile, Ser, Trp, Gln, Gly, Pro, Arg, Ala). Starting from the deviation distributions of these amino acids from the BLOSUM62 compositional background, we developed a scoring method to select those multiple alignments in BLOCKS data base whose sequences share the compositional bias of *Plasmodium *proteins. This approach assumes that those sequences and all *Plasmodium *proteins have similar background distributions and that the amino acid substitution frequencies observed in the alignments follow similar evolutionary rules and reflect those between *Plasmodium *proteins.

A vector (**T**) was constructed in which amino acids were represented as +1 or -1 values according to the direction of deviation. Similar vectors (**Bi**) were generated for each block in BLOCKS and then compared with the template by means of a scalar product (S = **T·Bi**). Three different parameters were introduced: S increases by 1 with each similar amino acid deviation shared between the template and the block; stringency was controlled by allowing up to 2 amino acids to be absent from a block (Z) and since tryptophan rarely occurs both in *Plasmodium *and BLOCKS sequences a third parameter (W) was introduced that allows this amino acid to be absent. Results of this scoring procedure are reported in table 1, where the numbers of blocks selected for each choice of S, Z and W are also shown.

A second scoring method calculates linear correlation coefficients (r) between the deviation distributions of *Plasmodium *proteins and those of sequences in each block of multiple alignments. According to this first method we selected three series of blocks; r > 0.50 (p < 0.05), r > 0.53 (p < 0.01), r > 0.70 (p < 0.001) (see table 1).

**Table 1:** Matrix generation criteria.

**Matrix**	**S**	**Z**	**W**	**r**	**B**
CBM/12-0-0	12	0	0	-	6
CBM/12-1-0	12	1	0	-	22
CBM/12-1-1	12	1	1	-	24
CBM/12-2-0	12	2	0	-	24
CBM/12-2-1	12	2	1	-	25
CBM/11-0-0	11	0	0	-	49
CBM/11-1-0	11	1	0	-	116
CBM/11-1-1	11	1	1	-	141
CBM/11-2-0	11	2	0	-	141
CBM/11-2-1	11	2	1	-	147
CBM/10-0-0	10	0	0	-	245
CBM/10-1-0	10	1	0	-	498
CBM/10-1-1	10	1	1	-	591
CBM/10-2-0	10	2	0	-	597
CBM/10-2-1	10	2	1	-	662
CBM/09-0-0	9	0	0	-	873
CBM/09-1-0	9	1	0	-	1505
CBM/09-1-1	9	1	1	-	1759
CBM/09-2-0	9	2	0	-	1784
CBM/09-2-1	9	2	1	-	1947
CCFMAT70	-	-	-	0.70	137
CCFMAT53	-	-	-	0.53	1196
CCFMAT50	-	-	-	0.50	1508

S = Required number of amino acids with expected deviation from BLOSUM62 values; Z = number of S amino acids allowed to be absent; W = value of one means tryptophan was allowed to be absent in addition to Z others; r = correlation coefficient lower limit; B = number of resultant blocks.

### Clustering procedure

For constructing the BLOSUM series of matrices, hierarchical clustering was used to group sequences of a given percentage identity before counting amino acid substitutions. This procedure reduces the contribution of closely related sequences to amino acid substitution counts as they are represented as a single cluster. A consequence of this is a loss of information, in fact, amino acid substitutions between sequences in the same cluster are not counted even if they do not share a level of similarity above the given threshold [1].

For these reasons, we decided to develop a non-hierarchical fuzzy clustering procedure that permits a sequence to be in more than one cluster and ensures that this sequence shares a given level of identity with all the other members in the same cluster, thus ensuring that all amino acid substitutions members are correctly counted. Fuzzy clustering algorithms have previously been used to represent the complex relationships between biological entities including amino acid sequences [18,19].

**Figure 2:**
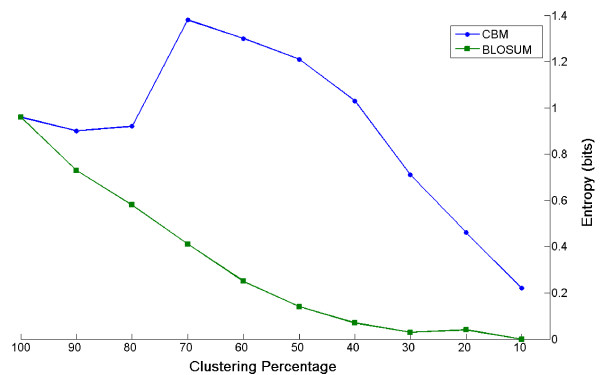
**Entropies of biased substitution matrices at different clustering percentages**. A series of substitution matrices were constructed from a subset of 1834 biased multiple alignments in BLOCKS data base. Sequences in multiple alignments were grouped by hierarchical (green line) and non-hierarchical clustering (blue line) procedures at different percentage of identity. For every matrix, the corresponding entropy value was calculated.

In order to study the effect of the two clustering procedures on matrix construction, we analysed the relationship between clustering percentage used to group sequences and the entropy associated with the resulting matrix. We selected a subset of 1834 biased multiple alignments from the BLOCKS database and generated substitution matrices using both hierarchical and our non-hierarchical clustering algorithm (see Methods for details). Substitution matrices were constructed by clustering sequences from 100% to 10% identity and for each matrix, the corresponding entropy was calculated (according to (7) in Methods). Matrices with identical entropies are generated when sequences are clustered at 100% (see figure 2), however while matrices constructed using hierarchical clustering have entropies which proportionally decrease with clustering percentage (green line in figure 2), matrices generated using our method show a different trend. Their entropies (see blue line in figure 2) first increase to a maximum at 70% and then decrease while always maintaining higher values than the corresponding matrices obtained by hierarchical clustering. This shows that non-hierarchical clustering is particularly suited to those cases in which the retention of information is crucial. The construction of matrices from compositionally biased sequences is one such case, due to the reduced number of blocks and the reduced substitution diversity within blocks. This is demonstrated by direct inspection of matrices. In fact, hierarchical clustering results in matrices with a peculiar pattern of high scores along the principal diagonal and mostly zero scores in the other cells while this is not observed in matrices obtained by means of our clustering method [see Additional file 2].

**Figure 3:**
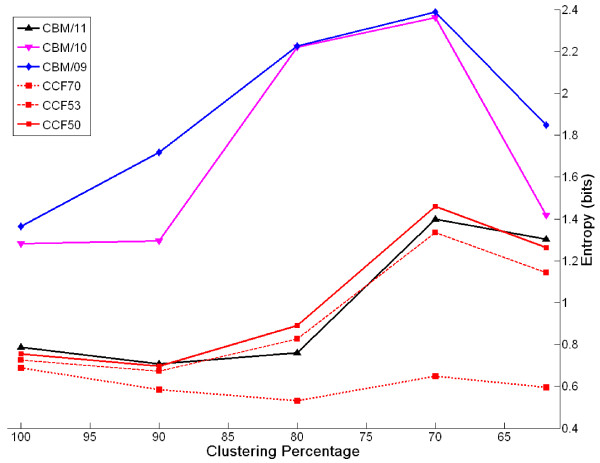
**Unconstrained entropies of CBM/CCF substitution matrices**. Novel series CBM/CCF of substitution matrices were constructed at different percentage of identity by a non hierarchical clustering procedure. Each series of matrices was constructed starting from subsets of compositionally biased multiple alignments from BLOCKS data base according to diverse selection criteria.

For these reasons, our fuzzy clustering method was adopted to group sequences in compositionally biased selected multiple alignments and hence to generate novel series of compositionally biased matrices. We constructed 23 matrices grouped into 5 series (see table 1) and for each of them we analysed the relationship of entropy to clustering percentage. The entropy trends observed for these matrices reflect those obtained from the previous analysis (see figure 3), apart from that obtained by using the CBM/12 matrices which are characterized by very low entropies resulting from the low number of blocks used to generate them (data not shown). In addition, we observed that a weak relaxation of selection criteria (varying Z and W parameters within the same CBM series; see table 1) does not imply a change in entropy values (in figure 3, the entropies of matrices belonging to the same CBM series are indistinguishable). Rather, when selection criteria are strongly relaxed (decreasing S parameter; see table 1) entropies of the matrices increase (see green, red and orange lines for CBM; see blue lines for CCF in figure 3) and reach maxima at 70% for CBM/10 and CBM/09. We established that this was due to the fact that few large multiple alignments contribute greatly to the counts of amino acid substitutions. A weak relaxation of selection criteria results in the addition of more, but shorter blocks that lead to slightly modified matrices.

**Figure 4:**
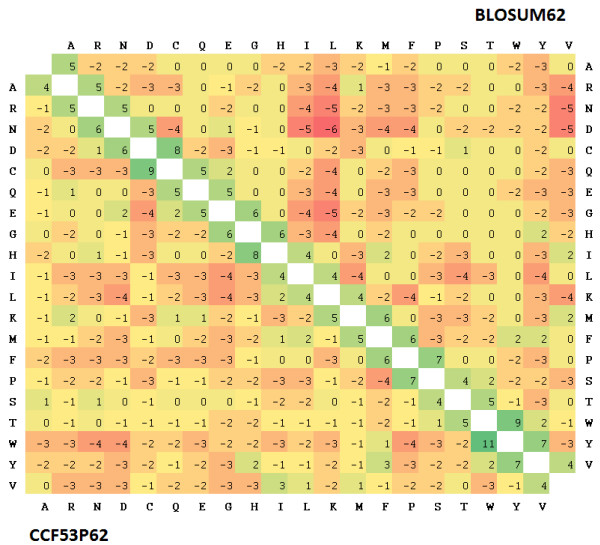
**Comparison of BLOSUM62 and CCF53_62 matrices**. The BLOSUM62 matrix is given on the upper right, while CCF53 is given on the lower left. Positive substitution scores are coloured green, while negative scores are red. Deeper colours represent more positive/negative scores.

As an example, in figure 4 scores for BLOSUM62 (upper right) and CCF53 clustered at 62% (lower left) are reported. As expected the scores along the principal diagonal remain similar between the two matrices. On the other hand, we observed that for certain amino acids which are strongly under-represented (i.e. W) or over-represented (i.e. N, K) in *Plasmodium *proteins, CCF53 substitution scores are far more negative and more positive respectively.

### Performance of matrices assessed by BLAST local alignments

In order to assess the performance of novel matrices, we compared results of BLAST database searches obtained by using CBM/09-2-1, CBM/10-2-1, CBM/11-2-1, CCF53 at 62% clustering, CBM/11-2-1 and CCF53 at 70% clustering (maximum entropy) and BLOSUM62 matrices (including the matrix compositional adjustment heuristic option of BLAST [6]). In order to negate the effect of entropy on matrix performance we constrained the entropy of each matrix to that of the BLOSUM62 matrix (Entropy = 0.69 ± 0.01 bits).

A unique database of all *P. falciparum *and *P. yoelii *proteins with an assigned gene ontology was then constructed, and all *P. falciparum vs *all *P. yoelii *BLAST searches were performed. Hits (with an associated e-value lower than 10^-10^) for each BLAST search were pooled and ranked by bit score, then GO identifiers of every pair of query and subject sequences were compared. Only hits between proteins sharing gene ontologies were considered as true positives (TP), while all other hits were considered false positives (FP). The numbers of false positives and true positives were reported as ROC_n _curves, and for every curve we calculated the area below (AUC_n_). This is an indicator of the matrix performance. Here, n was chosen to be 145 as this is the maximum number of false positives which are present in all searches that implied that not all hits for every database search were considered in this phase of assessment.

**Figure 5:**
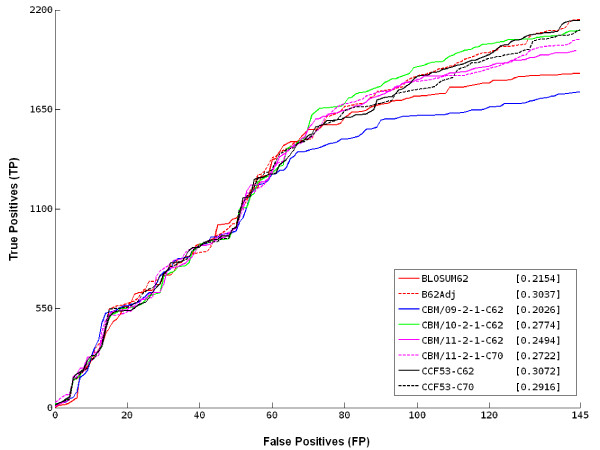
**ROC145 plot of PfGO vs. PyGO BLAST alignments**. ROC curves, at a cutoff of 145 false positives (FP), were calculated for several compositionally adjusted matrices and compared with BLOSUM62. In the key are reported AUC_145 _values corresponding to the area under ROC curves.

In general, the best performances (see AUC_145 _values in parentheses) are obtained by using CCF53 matrices (black lines in figure 5), CBM/10-2-1 (green line in figure 5) and the adjusted BLOSUM62 (dashed red line in figure 5). In more detail, all ROC_145 _curves in figure 5 are very alike in the initial regions, showing, as expected, that all examined matrices perform similarly when aligning highly similar proteins. In fact, the first part of the curves correspond to hits at the top of the ranked list and hence to pairs of sequences with high bit scores. However, in the latter region (after approximately 1300 TP hits), ROC_145 _curves diverge from each other. The number of false positive hits increases steeply for CBM/09-2-1 and BLOSUM62 while the other matrices show a less dramatic increase and thus better performance, in particular the matrix which works better in this region is CBM/10-2-1 as shown by the highest number of identified true positive hits.

These results were confirmed when we considered all hits for each database search and calculated other indicators of matrix performance, namely the positive predictive value (PPV = TP/(TP+FP) and the false discovery rate (FDR = FP/(FP+TP)) reported in table 2. The best performing matrices by these criteria are CBM/10-2-1 (PPV = 93.54%; FDR = 6.46%) and CCF53_62 (PPV = 93.11%; FDR = 6.89%) while results obtained by using the other novel matrices are comparable to those obtained by adjusted BLOSUM62.

**Table 2:** Hit statistics of matrices.

**Matrix**	**%ID**	**# hits**	**PPV (%)**	**FDR (%)**
BLOSUM62	62	4619	60.45	39.55
BLOSUM62Adj	62	2729	92.56	7.44
CBM/09-2-1	62	1981	91.92	8.08
CBM/10-2-1	62	2198	93.54	6.46
CBM/11-2-1	62	2406	91.44	8.56
CBM/11-2-1	70	2392	92.52	7.48
CCF53	62	2452	93.11	6.89
CCF53	70	2452	92.37	7.63

%ID = percentage clustering of selected blocks; PPV = TP/(TP+FP) positive predictive value; FDR = FP/FP+TP false discovery rate.

Our results confirm that the usage of compositionally modified matrices improve the identification of pairs of homologous proteins reducing the number of false positive hits. In addition we demonstrated that matrices constructed by us using the novel clustering procedure show a better performance even when compared with the compositional adjusted BLOSUM62.

### Biased matrices improve ortholog detection for hypothetical *Plasmodium *proteins

In a recent paper by Chen and colleagues [20], the performance of different methods of ortholog detection was assessed and it was shown that for homology based detection methods by BLAST the choice of e-value threshold has a strong effect on ortholog prediction error rates. As we have seen, our biased matrices result in better performance at an e-value of 1 × 10^-10^, however, we now wished to demonstrate the effects of the choice of e-value on homology detection. BLAST evalues can be considered accurate when proteins to be aligned have amino acid compositions which are not too dissimilar. We have previously shown that this is the case for examined *Plasmodium *proteins (PfH, PyH and PfO, PyO).

**Figure 6:**
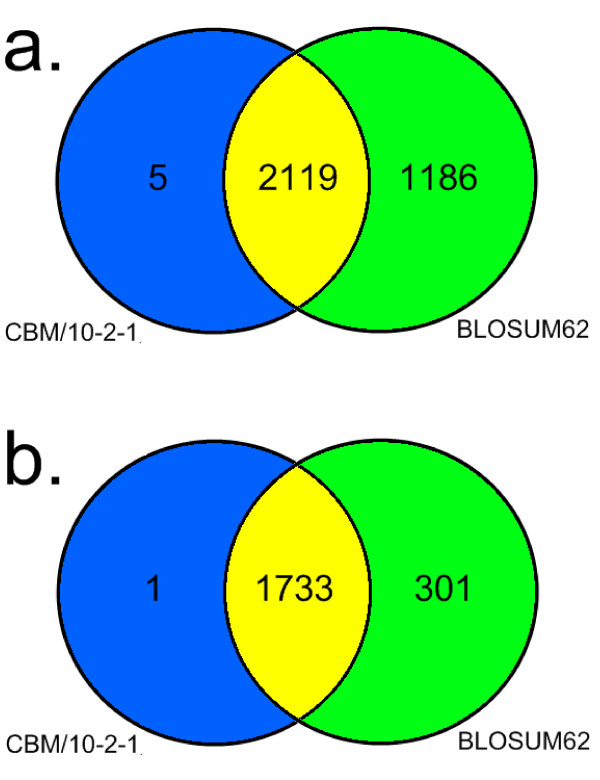
**Counts of P. falciparum/P. yoelii homologous pairs as identified using BLAST with different substitution matrices**. Results of an all vs. all BLAST search for *P. falciparum *and *P. yoelii *proteins are reported as Venn diagrams. Numbers correspond to the number of hits (homologs) found by using BLOSUM62 and CBM/10-2-1 matrices in BLAST at the two different e-value cutoffs 1e^-10 ^(A) and 1e^-20 ^(B). The number of hits identified by BLOSUM62 are in the green parts, that relative to hits identified only by CBM/10-2-1 are in the blue ones, while the amount of hits found by both matrices are in the yellow regions.

We considered proteins of *P. falciparum *and *P. yoelii *that are annotated as "hypothetical" (PfH and PyH). Such proteins constitute 60% of each proteome. Using BLAST, we aligned all 3385 *P. falciparum *hypothetical proteins with all 4576 *P. yoelii *hypothetical proteins and counted hits at two different e-value cutoffs. At the higher cut-off (e ≤ 1 × 10^-10^), we found that the use of CBM/10_2_1 (at 62% clustering) identified 2124 protein pairs while BLOSUM62 identified 3305 (see figure 6 panel A). Following from the observations that the usage of our matrices improves sensitivity of a BLAST database search (see previous section), we consider the extra 1186 hits found only by BLOSUM62 to be predominantly false positives. A case in point is that of MAL13P1.202. Twelve *P. yoelii *protein hits are identified using BLOSUM62, while using CBM/10-2-1, only the alignment with the candidate ortholog PY00593, assessed from genomic synteny mappings clears the threshold.

At the lower e-value cut-off (e ≤ 1 × 10^-20^), we found 2034 protein pairs using BLOSUM62 and 1734 using CBM/10-2-1 (figure 6 panel B). This represents a reduction in the number of hits by 38.4% and 18.4% respectively, when compared to hits obtained at the 1 × 10^-10 ^cut-off. This means that the choice of e-value cutoff has a less pronounced effect when using CBM/10-2-1 instead of BLOSUM62. Coupled with the increased specificity already demonstrated in the previous section, this suggests that novel matrices can help to improve ortholog detection by rendering it less sensitive to cutoff changes and more accurate at high cutoffs.

Interestingly, there are five protein pairs which were detected only by CBM/10-2-1 at a cutoff of 1 × 10^-10^. Of these, three pairs are candidate homologs, as evidenced by genomic synteny, while the other two proteins pairs cannot be evaluated due to lack of a detailed annotation. At a cutoff of 1 × 10^-20 ^a single further specific hit was found between PF14_0444 and PY01550 whose genes are in a syntenic region.

### Improving annotation of RIFIN/STEVOR proteins in *P*. *falciparum*

RIFIN/STEVOR genes are members of the most numerous multigene family in *P. falciparum *genome. The family numbers more than 190 genes that are almost exclusively found in sub-telomeric regions of chromosomes, organized in repeated tandem arrays [21]. Recent experimental data have shown that RIFIN and STEVOR products have different sub-cellular localization and are expressed in different stages of the parasite life cycle suggesting diverse but still unknown functions for these proteins [22]. Categorisation of members of this multigene family is a difficult task, as is highlighted by the presence of several proteins still annotated as "putative", by the changing annotations of several family members (for example, PFD0070c was initially annotated as a STEVOR, but then was changed to RIFIN; on the contrary annotation of PFD1220c and PFD0125c were changed from RIFIN to STEVOR) and by the shared InterPro domain (IPR002858: variant surface antigen RIFIN/STEVOR).

To assess the benefits of the use of compositionally adjusted matrices, we used both BLOSUM62 and our matrices to classify family members. 197 proteins annotated as RIFIN/STEVOR were downloaded from PlasmoDB [23] and *all vs. all *Needleman-Wunsch pairwise alignments [24] were performed using CCF53 and BLOSUM matrices at 62% clustering. A distance matrix was then built from similarity scores between each pair of proteins and this matrix was used as an input for classical multidimensional scaling (MDS) [25]. This well known statistical method creates a two-dimensional space in which Euclidean distances between points approximately reproduce those between sequences and thus allowed us to easily investigate similarity relationships between them.

**Figure 7:**
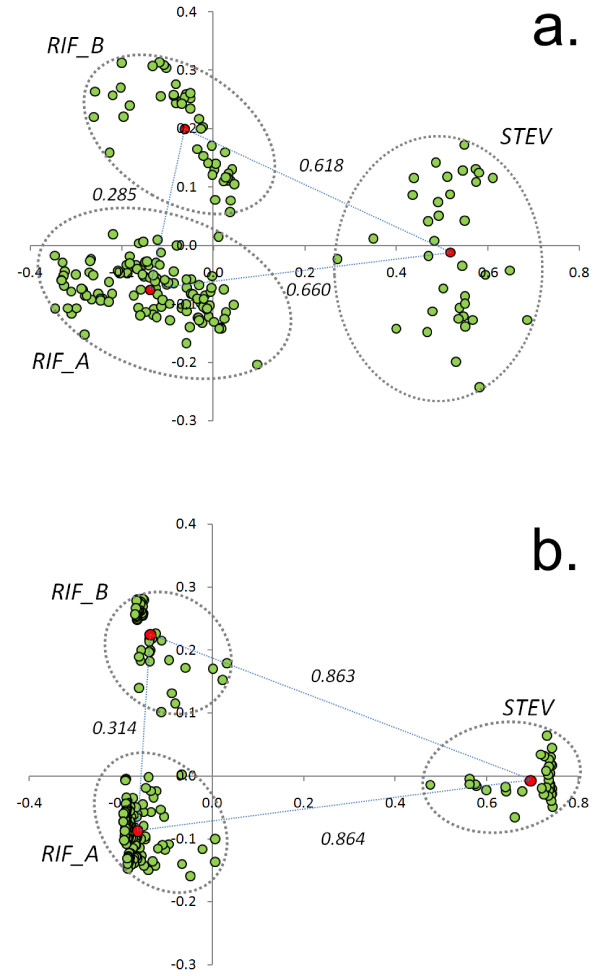
**Multi-dimensional scaling of RIFIN/STEVOR sequences**. Results of classical multidimensional scaling (MDS) of RIFIN/STEVOR protein sequences. Alignments of protein pairs were performed by Needleman-Wunsch algorithm by using both BLOSUM62 (A) and CCF53 (B). Corresponding percentages of similarity were used to calculate distance matrices that are used as input of MDS. Green dots represent sequences, red dots are the centroids calculated for each cluster (delimited by dashed ellipses). Distances between centroids (dotted lines) are also reported.

For MDS (figure 7) using both BLOSUM62 (panel A) and CCF53 (panel B), sequences (green dots) are grouped into three main clusters; one corresponds to the STEVOR sub-family while the others correspond to the RIFIN subfamilies, RIF_A and RIF_B [22,26]. Interestingly, however, when BLOSUM62 is used, clusters are not clearly defined making it difficult to assign sequences to a specific family. When CCF53 was used, clusters are more compact and definable and distances between clusters increase. Thus, the usage of novel matrices allowed us to distinguish clearly between subfamilies and clarify the classification of several borderline proteins (especially for RIF_A and RIF_B).

## Discussion

It is well recognised that dissimilar amino acid composition can affect alignments between proteins and as a consequence, can affect the accuracy of sequence similarity searches [4]. It thus holds, that in the case of compositionally biased proteomes, specific techniques should be adopted to maximise the protein annotation efficiency. This is particularly true for *P. falciparum *as it has been shown in several previous studies that the proteome is strongly biased towards certain amino acids [27-29]. In Pizzi et al. [30] it was shown that a large part of this bias is accounted for by frequent peculiar low-complexity sequences, characterised by a redundant usage of few amino acids. By assessing the compositional bias only on low-complexity filtered sequences, we carried out an analysis on proteins of both *P. falciparum *and *P. yoelii *and observed that, as expected, the compositional bias is strongly maintained in globular regions of these proteomes.

While many methods have been developed to date to deal with the issue of comparing proteins with a compositional bias, they have all been based on heuristic approaches [6,7] or on very specific protein sub-domains [5,9]. In this work, we wished to explore the possibility of empirically generating a series of substitution matrices which accurately represent substitution patterns between similarly biased proteins. To do this, we adjusted the method used for the generation of the BLOSUM substitution matrices. This empirical procedure is based on the amino acid substitution frequencies between sequences in multiple alignments (blocks) in BLOCKS data base. The crucial steps in the matrix generation are the choice of the subset of blocks and the clustering method used to remove redundant sequences within those blocks.

The first adjustment to the procedure was to use only those blocks with a compositional bias similar to the selected proteomes, making the assumption that the amino acid substitution rules are conserved throughout evolution. This eliminated between 96% and 99.9% of the blocks database, depending on the selection criteria, but allowed us to count substitution frequencies only in those protein domains with the desired bias.

The second adjustment was to develop a sequence clustering method which allowed us to maintain a complete representation of substitution frequencies while removing bias due to highly similar sequences. Classical procedures such as hierarchical and single-linkage clustering (see [31] for an application to non-redundant sequences selection) are not able to provide a complete representation of the complex network of sequence relationships. The main limitation resides in the requirement for a sequence to be member of a single cluster. Pyramidal clustering [32] is an example of how to effectively overcome this problem, permitting a sequence to be in up to two clusters. In the case of our algorithm we followed a similar approach, however our clustering procedure allows sequences to belong to multiple clusters (more than two), and ensures that all members of a cluster share a given similarity. We showed that this fuzzy clustering was important in the case of biased matrices as it allowed us to maintain sufficient substitution information to construct matrices solely from biased blocks.

In the absence of a *bone fide *test dataset of similarly biased proteins, the use of GO allowed us to assess the accuracy of protein alignments. For this reason, we first considered all *P. falciparum *and *P. yoelii *proteins for which gene ontology (GO) annotations are available. A possible flaw in this assessment criteria is that GO annotations are sometimes assigned solely as a result of sequence similarity between proteins. As this is only done when there is a very high level of homology incorrect GO assignments should be minimal. We aligned by BLAST, all selected *P. falciparum *proteins with all selected *P. yoelii *proteins using our matrices, BLOSUM62, and a heuristic BLAST method published recently based on a compositional adjustment of BLOSUM62 [6]. Comparison with this heuristic BLOSUM adjustment serves as a reference since it was already shown to improve alignments of biased proteins. We obtained the best results by using matrices CCF53 and CBM/10-2-1. These outperformed both BLOSUM62 and adjusted BLOSUM62 in BLAST searches. The use of all of our matrices resulted in a dramatic reduction in false positive (incorrect) alignments with respect to BLOSUM62, and in all cases yielded a similar improvement to that achieved using the compositional adjustment BLAST heuristic. Furthermore, higher bit scores and e-values, and lower lengths for true positive hits were found by using CCF/CBM instead of BLOSUM matrices. To explain these results, we must consider the way in which the Smith-Waterman algorithm works. In the scoring matrix constructed by dynamic programming, local similarities are identified as continuous diagonals flanked by cells with zero values. Starting from the highest scoring similar regions, substitution scores in BLOSUM62 facilitate the elongation of diagonals, thereby extending the alignments and often decreasing the global bit-scores. On the other hand, lower substitution scores in CCF/CBM matrices impede the elongation process, and in case of low similarity between sequences do not allow extension of diagonals which may correspond to unreliable alignments.

Having established the better performance of our matrices with respect BLOSUM series, we investigated the possibility of using them to improve annotation of *Plasmodium *proteins for which orthologs in other organisms are not known and hence are potentially specific for the parasites. We used CBM/10-2-1 to identify homologs between *P. falciparum *and *P. yoelii *proteins that are annotated as "hypothetical". Since these and more frequent low-complexity sequences we would expect that number of possible false positive hits to increase greatly and hence, make the identification of homologs particularly difficult. Our matrices identify far less putative homologs than BLOSUM62, and also yield a more consistent result set at an e-value of 1 × 10^-10 ^(when compared with an e-value of 1 × 10^-20^).

As the e-value accuracy has been shown to be an important criterion for ortholog annotation [20], the increased alignment confidence at low e-values using our matrices will improve the initial step of the OrthoMCL algorithm [33] on which the current ortholog mappings for *Plasmodia *are based.

We finally showed a non-BLAST based application of our method. The accurate annotation of proteins belonging to RIFIN/STEVOR family represents a difficult task because of the sequence variability between members. Although more than 197 proteins are assigned to this family, evidence is emerging that they have many and diverse sub-cellular localization, differential expression patterns and hence probable diverse functions [34]. Furthermore it was established that STEVORs constitute a small distant subset of proteins while RIFINs can be divided into two subgroups of proteins [13,34]. Global alignments using the Needleman-Wunsch algorithm were performed between all proteins annotated as RIFIN/STEVOR using both CCF53 and BLOSUM62 matrices. The derived global similarity percentages were thus used to construct distance matrices. In order to explore relationships among members of the family, we performed multidimensional scaling that provides a simple visualisation of sequences as points in a two dimensional space.

When we used CCF53, instead of BLOSUM62, distances between sub-families (STEVOR, RIF_A and RIF_B) are much larger and thus a clear classification of the proteins into distinct families was obtained, especially in the case of RIF_A and RIF_B. Interestingly, by this method we can also identify the more likely evolutionary links between these gene families as those proteins which remain near the periphery of clusters.

## Conclusion

In this work, we illustrated the role played by amino acid distributions in the accuracy of protein alignments between two related species which have similarly skewed background amino acid compositions. We showed that a novel series of compositionally adjusted matrices are better suited to compare diverse classes of *Plasmodium *proteins. When CBM/CCF matrices are used a reduction in the number of false positive hits is observed, more consistent results in the identification of homologs between *Plasmodium *species are obtained and a clearer classification for members of a multigene families is achieved.

The use of these matrices will increase confidence in the annotation of the proteins from these organisms and from others which share a similar amino acid compositional bias. This will further permit a refined analysis of polymorphisms at the amino acid level which will be of considerable interest given the imminent sequencing of a large number of *Plasmodium *genomes, including *P. falciparum *field isolates.

## Methods

### Data Sets

*P. falciparum *and *P. yoelii *sequence data were downloaded from PlasmoDB v5.0 [35]. For each set of sequences, we selected those for which an annotated ortholog existed in a non-*Plasmodium *species (PfO, PyO), and proteins classified as hypothetical without orthologs in other non-Plasmodia (PfH, PyH).

### Scoring blocks

The amino acid background frequencies implicit in the BLOSUM62 matrix were derived from a version of the BLOSUM62 matrix, scaled by a factor of 151.9 and using the Newton_bioinfo program of Yu et al. [4]. *P. falciparum *and *P. yoelii *proteins in the four data sets PfO, PyO, PfH and PyH were filtered using SEG program [36] (window size 24; locut 2.3; hicut 2.4), then for each data set the percent divergence of each amino acid from the BLOSUM62 value (d) was calculated according to (1).

(1) $$d=\frac{f(a_{i})-f(a_{b62})}{f(a_{b62})}\cdot 100$$

where f(a_i_) and f(a_b62_) are the frequency of amino acid i in the data set and the background frequency implicit in BLOSUM62 respectively.

Similar distributions of amino acid deviations were generated for all 28337 blocks of multiple alignments in the BLOCKS database (version 14.1) [15,16,37], then we adopted two different methods to select blocks of multiple alignments with amino acid deviations compatible with that of *Plasmodium *proteins.

Firstly blocks were selected that correlate to *P. falciparum *(PfO) deviations according to the Pearson s correlation coefficient.

The second method only considered those amino acids that deviate by more than 20% in at least one of the two datasets of both *P. falciparum *and *P. yoelii *proteins. These were identified as Pro, Arg, Ala, Trp, Gly, Ile, Asn, Lys, Tyr, Glu, Gln, Ser. A template was generated as a vector whose directions are +1 or -1 (**T**) according to the direction of compositional bias, that is **T **(-1, -1, -1, -1, -1, +1, +1, +1, +1, +1, -1, +1). The amino acid distributions of each multiple alignment in the BLOCKS database were similarly vectorised (**B**) and a score (S) was calculated as the scalar product of **T **and **B**. In this way for each amino acid for which the deviation from BLOSUM62 was in agreement with the template, the S score was incremented by 1 with the maximum equal to 12. In order to select a higher number of blocks, we introduced two parameters to allow amino acids to be absent from blocks, Z for any amino acid (up to 2 amino acids in one block) and W for tryptophan. Z and W can assume value 0, 1, 2 and 0, 1 respectively, according to the number of missing amino acids.

### Matrix generation

For each series of selected blocks, log odds matrices were generated. For BLOSUM matrices, hierarchical clustering was used to avoid over-counting due to similar sequences within blocks of multiple alignments. In order to retain maximum sequence information, we applied a non-hierarchical fuzzy clustering method which allowed a sequence to participate in more than one cluster, and guaranteed that amino acid substitutions for every pair of sequences not sharing a given percentage identity were counted.

For each block, a matrix of percent identity scores between sequences (d_xy_) was constructed. For a given threshold of similarity and starting from the first sequence (s_a_), the matrix was traversed vertically until a sequence, s_x_, with d_ax _>= threshold was encountered. s_x _was then clustered with s_a_. This column was traversed completely, adding sequences (s_z_) to the cluster which had d_xz _>= threshold for all other member sequences. Sequences for which d_az _>= threshold, but d_xz _< threshold for other members, initialise new clusters which were populated using the same procedure. Sequences already assigned to a cluster are not excluded by this iteration and in this way can take part in other clusters. Once all combinations for this column of the matrix were examined, the procedure re-initiated from the top of the next column.

Log odds substitution scores were then generated using the formula:

(2) $$S_{ij}=\frac{1}{\lambda }\log_{2}\left(\frac{q_{ij}}{p_{i}p_{j}}\right)$$

where q_ij _are the observed frequencies of amino acid substitutions, and p_i_, p_j _are the expected frequencies of individual amino acids. q_ij _were calculated as

(3) $$q_{ij}=\frac{f_{ij}}{\sum_{i=1}^{20}\sum_{j=1}^{i}f_{ij}}$$

According to the proposed clustering procedure, f_ij _scores were calculated in the following way. For each sequence which participates in n clusters, n diverse weights were calculated according to:

(4) $$w_{i}=\frac{1/n_{i}}{\sum_{i=1}^{N}1/n_{i}}$$

where N is the number of sequences in the i^th ^cluster, and n_i _is the number of clusters in which a sequence participates. We were thus able to weight each amino acid in each column and to calculate amino acid pair counts by:

(5) f_ij _= ∑_i_∑_j _w_ix_·w_jy _    for x ≠ y

For each amino acid pair the final count is:

(6) F_ij _= ∑_b_∑_p _f_ij_

p = 1,... L where L is the length of a multiple alignment in a block and b = 1,... T where T is the number of blocks. The target (q_ij_) and background (p_i_) frequencies for each set of blocks were then derived.

A matrix was then created, and the expected score and un-gapped entropy (H_u_) of each matrix was calculated.

(7) H_u _= ∑_i = 1 _∑_j = i _q_ij_·s_ij_

The statistical parameters, lambda, K, H, alpha and beta were derived for each matrix using affine gap penalties of (-11:-2) using the Island Method [38]. Version 2.2.10 of BLASTP [39], contained in the NCBI toolkit build 06/12/2005, was obtained from the NCBI website [40]. Two files were altered, blastkar.c and blast_stat.c to incorporate the statistical details of the new matrices.

To assess the effects of our fuzzy clustering method on matrix entropy, we generated matrices clustered at 100%, 90%, 80%, 70%, 60%, 50%, 40%, 30%, 20% and 10% from the same set of biased multiple alignments using both our algorithm and the BLOSUM algorithm [41]. The multiple alignments used were those selected using the CBM/09-2-1 criteria (table 1). Due to limitations of the BLOSUM algorithm, only multiple alignments less than 10 kilobytes in size were used. This resulted in 1834 multiple alignments and the exclusion of 113 larger ones.

### Restricting matrix entropy

In order to negate the implicit effects of matrix entropy on alignments, all matrices were scaled to have a entropy of 0.69 ± 0.01 bits (entropy of BLOSUM62). In the case of CBM/10-2-1 and CBM/09-2-1, for all possible affine gap penalty combinations for BLOSUM62, statistical values for BLAST could not be calculated using the island method. These were thus excluded from further analysis.

### Matrix performance assessment

Two group of sequences were obtained by selecting 1687 *P. falciparum *and 1650 *P. yoelii *proteins for which a reliable annotation and hence, a gene ontology (GO) identifier was assigned. Gene ontologies and *P. falciparum *GO annotations were downloaded from the Gene Ontology website [42] while *P. yoelii *GO annotations were obtained from EBI [43]. We considered only GO terms for molecular function and biological process and excluded proteins for which the only GO terms were top level generic. The two groups of sequences were used to construct two databases (*P. falciparum *GO (PfGO) and *P. yoelii *GO (PyGO)). For each matrix, an all PfGO against all PyGO BLASTP search was performed. For this we used BLAST 2.2.10, modified as described in the previous section to include the statistics for the new matrices. In BLASTP searches, we used an e-value threshold < 1e^-05^, with a maximum of 250 hits for any query-subject pair. The -t 2 parameter was used with blastpgp for compositional adjustment of BLOSUM62, and in this paper, BLAST searches using this parameter will be referred to as B62Adj. In all cases, results were collected and hits ranked by e-value. Hits were then classified as true positives if they were annotated with a common GO or false positives if there was no common GO for the pair of proteins.

### Multidimensional scaling of RIFIN/STEVOR protein sequences

All *P. falciparum *proteins containing the InterPro domains IPR006373 or IPR006374 or the PFAM domain PF02009 were obtained from PlasmoDB. Pseudogene products were excluded from the dataset. This resulted in 197 proteins. We then performed global alignments of each of these proteins against all others in the dataset using the NEEDLE algorithm of EMBOSS 5.0.0 [24]). A percentage similarity distance matrix was then generated and used as input for classical multidimensional scaling (Torgerson-Gower scaling [25]).

### Software implementation

The clustering and matrix generation algorithms were developed in perl and implemented in a Windows XP environment. Statistics for each matrix were calculated using the Island Method C++ code provided by Stephen Altschul in fedora core 3 linux. BLAST was modified and recompiled in this environment. Complex queries and Gene Ontology distance analysis were performed using Microsoft Visual FoxPro 8.0. Work was performed on a Pentium 4 dual processor PC with 4GB RAM.

## Authors' contributions

KB carried out calculations and data analysis. KB and EP participated in the design and coordination of the study, the analysis of results and the writing of the manuscript. All the authors have read and approved the final version of the manuscript.

## Supplementary Material

Additional file 1 AA_Distributions.pdf, pdf, Absolute amino acid frequencies in *Plasmodium *proteins Click here for file

Additional file 2 BLOSUM VS CBM Matrices.xlsx, xlsx, The effect of information loss from clustering on matrices built from biased blocks Click here for file
