# Supplementary figures and images for: A novel series of compositionally biased substitution matrices for comparing Plasmodium proteins
Source: BMC Bioinformatics. 2008 May 16;9:236. doi: 10.1186/1471-2105-9-236 (PMC2408606; doi:10.1186/1471-2105-9-236)

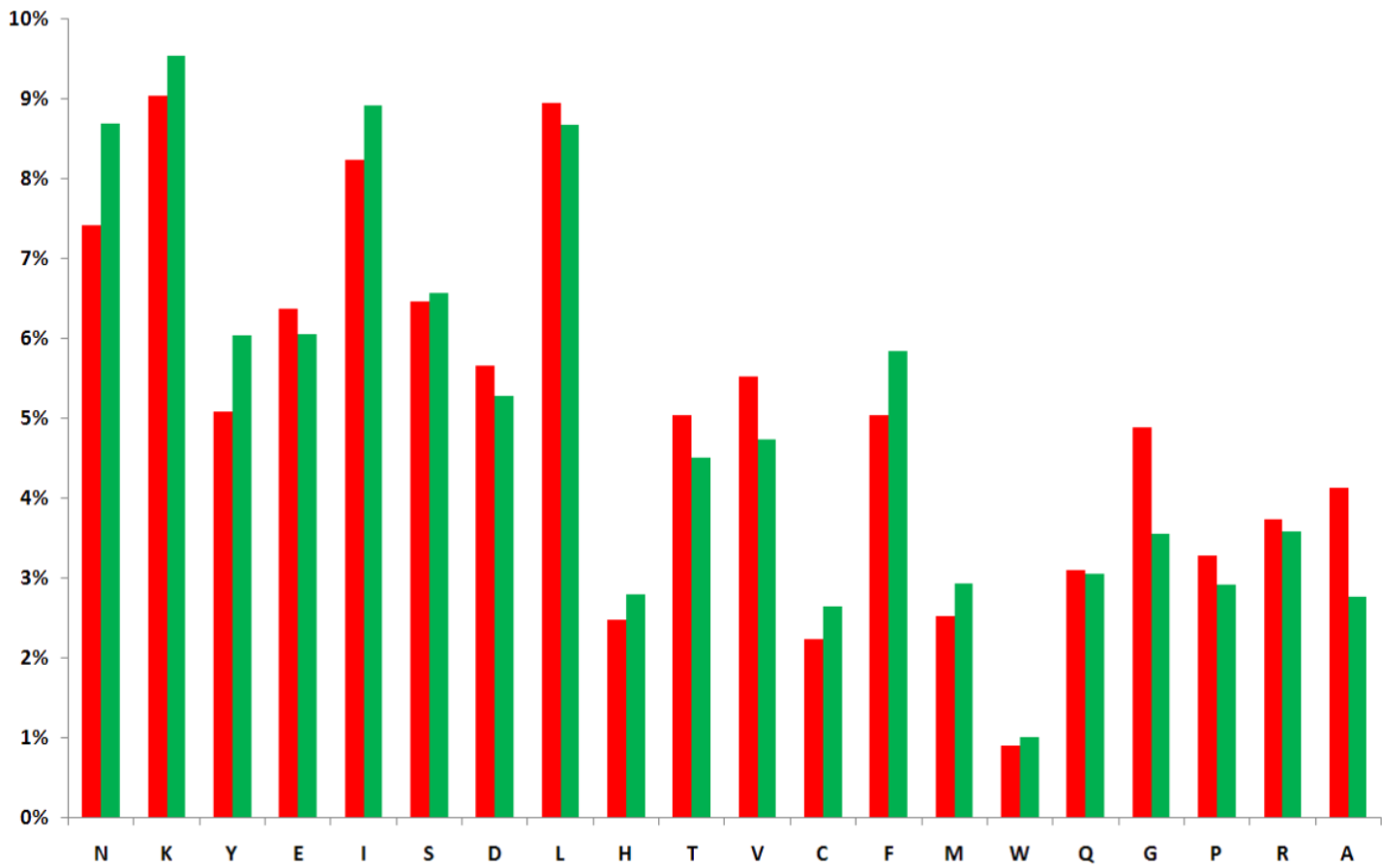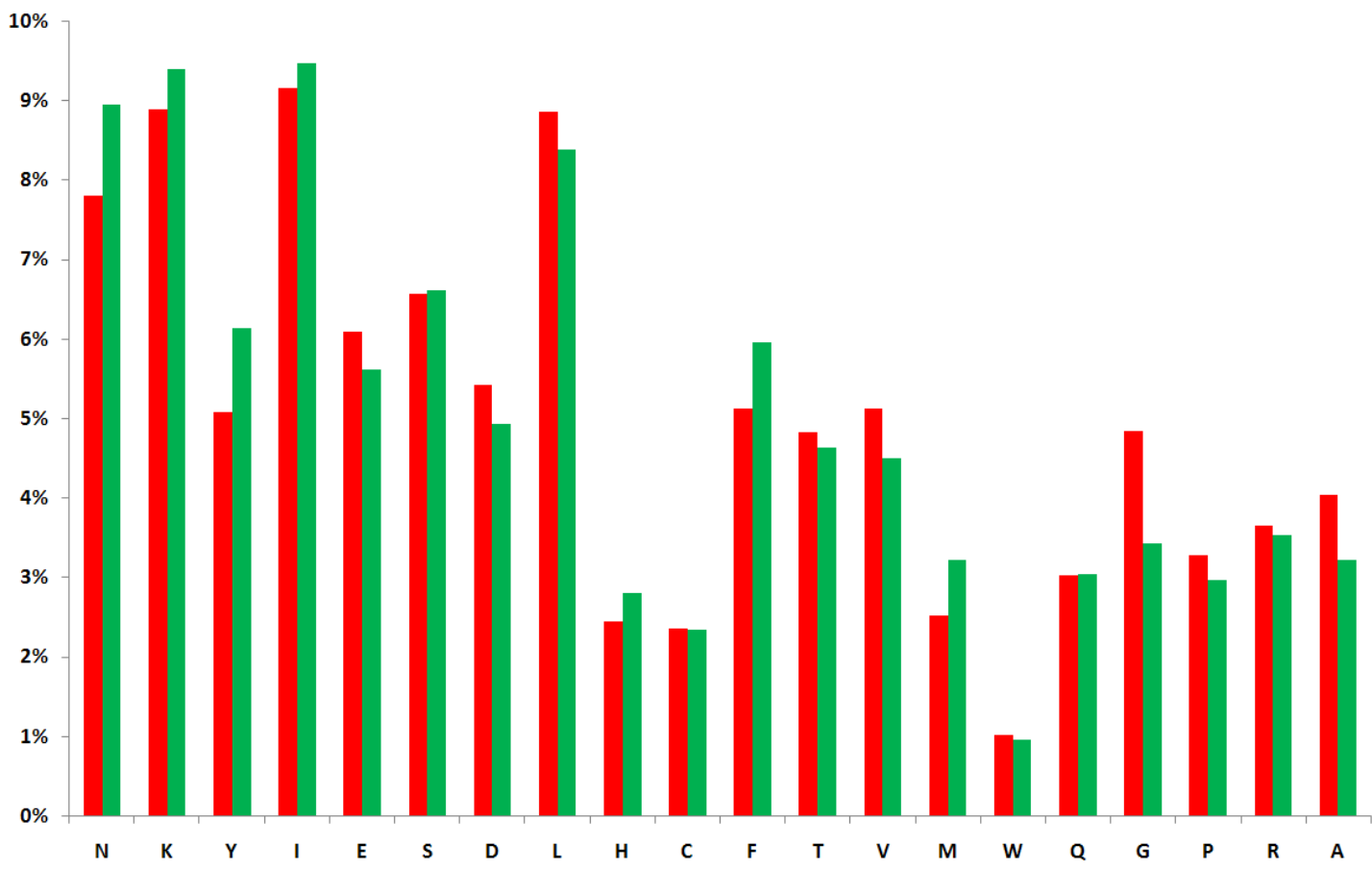

Supplement: Additional file 1 — AA_Distributions.pdf, pdf, Absolute amino acid frequencies in Plasmodium proteins [file 1471-2105-9-236-S1.pdf]
